# Ternary Copper(II) Complexes in Solution^[1,2]^ Formed With 8-Aza Derivatives of the Antiviral Nucleotide Analogue 9-[2-(Phosphonomethoxy)Ethyl]Adenine (PMEA)

**DOI:** 10.1155/MBD.2000.313

**Published:** 2000

**Authors:** Raquel B. Gómez-Coca, Larisa E. Kapinos, Antonín Holý, Rosario A. Vilaplana, Francisco González-Vílchez, Helmut Sigel

**Affiliations:** 1 Institute of Inorganic Chemistry University of Basel Spitalstrasse 5 l Basel CH-4056 Switzerland; 2 Inorganic Chemistry Department Faculty of Chemistry University of Seville Seville E-41071 Spain; 3 Institute of Organic Chemistry and Biochemistry Academy of Sciences Prague CZ-16610 Czech Republic

## Abstract

The stability constants of the mixed-ligand complexes formed between Cu(Arm)^2+^, where Arm= 2,2'-bipyridine (Bpy) or 1,10-phenanthroline (Phen), and the dianions of 9-[2-(phosphonomethoxy)ethyl]-8-azaadenine (9,8aPMEA) and 8-[2-(phosphonomethoxy)ethyl]-8-azaadenine (8,8aPMEA) (both also abbreviated as PA^2-^) were determined by potentiometric pH titrations in aqueous solution (25 ^°^C; *I* = 0.1 M, NaNO_3_). All four ternary Cu(Arm)(PA) complexes are considerably more stable than corresponding Cu(Arm)(R-PO_3_) species, where R-PO_3_^2-^ represents a phosph(on)ate ligand with a group R that is unable to participate in any kind of interaction within the complexes. The increased stability is attributed to intramolecular stack formation in the Cu(Arm)(PA) complexes and also to the formation of 5-membered chelates involving the ether oxygen present in the -CH_2_-O-CH_2_-PO_3_^2-^ residue of the azaPMEAs. A quantitative analysis of the intramolecular equilibria involving three structurally different Cu(Arm)(PA) species is carried out. For example, about 5% of the Cu(Bpy)(8,8aPMEA) system exist with the metal ion solely coordinated to the phosphonate group, 14% as a 5-membered chelate involving the -CH_2_-O-CH_2_-PO_3_^2-^residue, and 81% with an intramolecular stack between the 8-azapurine moiety and the aromatic rings of Bpy. The results for the other systems are similar though with Phen a formation degree of about 90% for the intramolecular stack is reached. The existence of the stacked species is also proven by spectrophotometric measurements. In addition, the Cu(Arm)(PA) complexes may be protonated, leading to Cu(Arm)(H;PA)^+^ species for which it is concluded that the proton is located at the phosphonate group and that the complexes are mainly formed by a stacking adduct between Cu(Arm)^2+^ and H(PA)^-^. Conclusions regarding the biological properties of these azaPMEAs are shortly indicated.


### TERNARY COPPER(II) COMPLEXES IN SOLUTION[1,2] FORMED WITH 8-AZA DERIVATIVES OF THE ANTIVIRAL NUCLEOTIDE ANALOGUE 9-[2-(PHOSPHONOMETHOXY)ETHYL]ADENINE (PMEA)

Raquel B. Gómez-Coca[1,2], Larisa E. Kapinos[1], Antonín Holý[3], Rosario A. Vilaplana[2], Francisco González-Vílchez[2] and Helmut Sigel*[1]

[1] Institute of Inorganic Chemistry, University of Basel, Spitalstrasse 51, CH-4056 Basel, Switzerland <Helmut.Sigel@unibas.ch>
[2] Inorganic Chemistry Department, Faculty of Chemistry, University of Seville, E-41071 Seville, Spain
[3] Institute of Organic Chemistry and Biochemistry, Academy of Sciences, CZ-16610 Prague, Czech Republic

*Dedicated to the memory of Professor Marc Leng, an outstanding scientist and friend*

**Abstract**

The stability constants of the mixed-ligand complexes formed between $Cu(Arm)^{2+}$, where Arm = 2,2'-bipyridine (Bpy) or 1,10-phenanthroline (Phen), and the dianions of 9-[2-(phosphonomethoxy)ethyl]-8-aza-adenine (9,8aPMEA) and 8-[2-(phosphonomethoxy)ethyl]-8-azaadenine (8,8aPMEA) (both also abbreviated as $PA^{2-}$) were determined by potentiometric pH titrations in aqueous solution (25 °C; $I$ = 0.1 M, $NaNO_3$). All four ternary Cu(Arm)(PA) complexes are considerably more stable than corresponding $Cu(Arm)(R\text{-}PO_3)$ species, where $R\text{-}PO_3^{2-}$ represents a phosph(on)ate ligand with a group R that is unable to participate in any kind of interaction within the complexes. The increased stability is attributed to intramolecular stack formation in the Cu(Arm)(PA) complexes and also to the formation of 5-membered chelates involving the ether oxygen present in the $\text{-}CH_2\text{-}O\text{-}CH_2\text{-}PO_3^{2-}$ residue of the azaPMEAs. A quantitative analysis of the intramolecular equilibria involving three structurally different Cu(Arm)(PA) species is carried out. For example, about 5% of the Cu(Bpy)(8,8aPMEA) system exist with the metal ion solely coordinated to the phosphonate group, 14% as a 5-membered chelate involving the $\text{-}CH_2\text{-}O\text{-}CH_2\text{-}PO_3^{2-}$ residue, and 81% with an intramolecular stack between the 8-azapurine moiety and the aromatic rings of Bpy. The results for the other systems are similar though with Phen a formation degree of about 90% for the intramolecular stack is reached. The existence of the stacked species is also proven by spectrophotometric measurements. In addition, the Cu(Arm)(PA) complexes may be protonated, leading to $Cu(Arm)(H;PA)^+$ species for which it is concluded that the proton is located at the phosphonate group and that the complexes are mainly formed by a stacking adduct between $Cu(Arm)^{2+}$ and $H(PA)^-$. Conclusions regarding the biological properties of these azaPMEAs are shortly indicated.

### 1. INTRODUCTION

Nucleotides and their metal ion complexes play a key role in all aspects of metabolism and therefore, attempts to exploit nucleotide analogues as drugs are old (e.g.[3]). Among the analogues with biological properties 9-[2-(phosphonomethoxy)ethyl]adenine (PMEA),[4] an analogue of (2'-deoxy)-adenosine 5'-monophosphate [(d)$AMP^{2-}$], is a most remarkable one; it exhibits antiviral,[5-8] cytostatic[9,10] and antiarthritic[11] effects.

Considering the broad biological activity of PMEA, it is not surprising that many derivatives have been synthesized and studied,[12] and it is now clear that in order to be antivirally active, PMEA and its derivatives must be phosphorylated in the cell to the diphosphate ($PMEApp^{4-}$) and this is then recognized by DNA polymerases as a substrate and incorporated into the growing nucleic acid chain which is terminated thereafter.[13,14] Knowing that polymerases depend on the presence of metal ions[15,16] and that the nucleoside 5'-triphosphates must be present as complexes (mostly $Mg^{2+}$)[17] we have been studying complexes of PMEA[18-24] and a mechanism of action has been proposed recently[25,26] in which the correct location of two metal ions at the triphosphate chain for achieving a desired reaction is emphasized.[26] In other words, the correct orientation of the nucleoside 5'-triphosphate or its analogue in the active-site cavity of the enzyme is crucial.[26]

One of the ways in which the correct anchoring process of a substrate in the active site of an enzyme can be achieved, is *via* stacking interactions[27] of the nucleobase residue, e.g. with an indole moiety of a tryptophan unit.[28] For this reason we became interested in the PMEA relatives, 9-[2-(phosphonomethoxy)ethyl]-8-azaadenine (9,8aPMEA) and 8-[2-(phosphonomethoxy)ethyl]-8-azaadenine (8,8aPMEA) (see Fig. 1), and the question was: Do the stacking properties of PMEA, 9,8aPMEA and 8,8aPMEA differ? To this end we measured the stabilities of the mixed ligand Cu(Arm)(PA) complexes, where Arm = 2,2'-bipyridine (Bpy) or 1,10-phenanthroline (Phen) and $PA^{2-}$ = $9,8aPMEA^{2-}$ or $8,8aPMEA^{2-}$. These Cu(Arm)(PA) complexes can fold such that the aromatic rings of Bpy or Phen can interact with the 8-azaadenine residues. In fact, Bpy and Phen have proven very helpful as indicators for evaluating the stacking capabilities of aromatic residues in metal ion complexes.[28] A stacking interaction of the indicated kind should be reflected in an enhanced overall complex stability.[2,29,30] Indeed, the results obtained prove such enhanced stabilities and these are compared now with those obtained earlier[31] for the corresponding Cu(Arm)(PMEA) complexes.

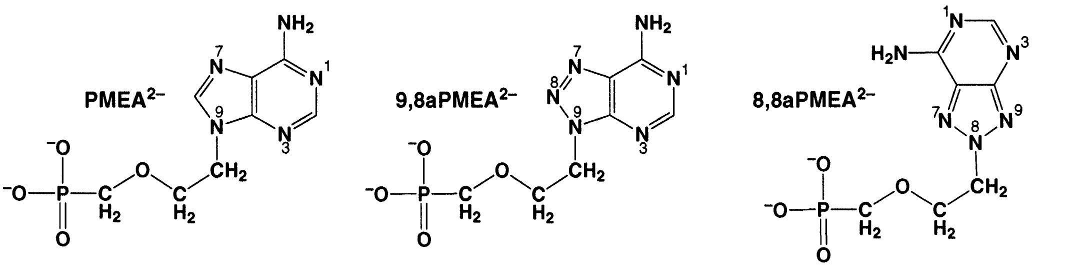

**Figure 1.** Chemical structures of the dianions of 9-[2-(phosphonomethoxy)ethyl]adenine ($PMEA^{2-}$), 9-[2-(phosphonomethoxy)ethyl]-8-azaadenine ($9,8aPMEA^{2-}$) and 8-[2-(phosphonomethoxy)ethyl]-8-azaadenine ($8,8aPMEA^{2-}$). The three nucleotide analogues are also abbreviated as $PA^{2-}$.

### 2. MATERIALS AND METHODS

### *2.1. Materials*

Twofold protonated 9-[2-(phosphonomethoxy)ethyl]-8-azaadenine, *i.e.* $H_2(9,8aPMEA)^{\pm}$, and its 8-isomer 8-[2-(phosphonomethoxy)ethyl]-8-azaadenine, $H_2(8,8aPMEA)^{\pm}$, were synthesized by alkylation of 8-azaadenine with a synthon carrying the structural features of the required side chain.[32] 2,2'-Bipyridine, 1,10-phenanthroline monohydrate, and the nitrate salts of $Na^+$ and $Cu^{2+}$ (all *pro analysi*) were from Merck AG, Darmstadt, FRG. All the other reagents were identical with those used previously[24] and all solutions for the potentiometric pH titrations were prepared with ultrapure $CO_2$-free water as described.[24]

### *2.2. Potentiometric pH Titrations*

The apparatus for the potentiometric pH titrations, the calibration procedure, the computers, and the calculation methods used now are the same as in [24]. The stability constants $K^M_{M(H;9,8aPMEA)}$ and $K^M_{M(9,8aPMEA)}$, where $M^{2+}$ = $Cu(Bpy)^{2+}$ or $Cu(Phen)^{2+}$, were determined by titrating 30 mL of aqueous 0.83 mM $HNO_3$, 0.4 mM $9,8aPMEA^{2-}$, and 4.4 mM or 2.2 mM $Cu^{2+}$/Arm (*i.e.*, $Cu^{2+}$:Arm:$PA^{2-}$ = 11:11:1 or 5.5:5.5:1) under $N_2$ (25 °C; $I$ = 0.1 M, $NaNO_3$) with 1 mL 0.03 M NaOH. Each titration was repeated in the absence of ligand and the differences in NaOH consumption between such a pair of titrations were used for the calculations.

The conditions for the measurements with 8,8aPMEA were identical with those given above for 9,8aPMEA. It may be added that the acidity constants $K^H_{H_2(PA)}$ and $K^H_{H(PA)}$ for $H_2(PA)^{\pm}$ and $H(PA)^-$, respectively, and the stability constants $K^{Cu}_{Cu(H;PA)}$ and $K^{Cu}_{Cu(PA)}$ for the binary $Cu(H;PA)^+$ and Cu(PA) complexes were determined under the corresponding conditions.[33] Furthermore, the above conditions are similar to those described in [24].

Under the given experimental conditions the formation of the $Cu(Arm)^{2+}$ complexes is practically complete[34] (in agreement herewith, titrations of solutions with $HNO_3$ and $HNO_3$ plus $Cu^{2+}$/Arm were identical in the lower pH range) and therefore, the evaluation of the titration data of the ternary complexes could be done in the way described previously for binary complexes.[24] The $Cu(Bpy)^{2+}$/9,8aPMEA 5.5:1 and 11:1 systems were evaluated in the pH range 3.4-5.3, reaching formation degrees of about 4.5 and 8% for $Cu(Bpy)(H;9,8aPMEA)^+$ and 64 or 78% for Cu(Bpy)(9,8aPMEA), respectively. For the $Cu(Phen)^{2+}$/9,8aPMEA 5.5:1 and 11:1 systems data were collected in the pH ranges 3.4-5.0 and 3.4-4.8, respectively, reaching formation degrees of about 8 and 14% for $Cu(Phen)(H;9,8aPMEA)^+$ and about 61 or 66% for Cu(Phen)(9,8aPMEA). The upper limits of the evaluated pH ranges were always determined by the beginning of the hydrolysis of the $Cu(Arm)^{2+}_{aq}$ species. Similarly, the $Cu(Bpy)^{2+}$/8,8aPMEA 5.5:1 and 11:1 systems were evaluated between pH 3.6-5.3 and 3.5-5.3, respectively, with formation degrees of about 7 and 12% for $Cu(Bpy)(H;8,8aPMEA)^+$ and about 62 or 76% for Cu(Bpy)(8,8aPMEA), respectively. For the $Cu(Phen)^{2+}$/8,8aPMEA 5.5:1 and 11:1 systems data were collected in the pH ranges 3.6-5.0 and 3.5-5.0, respectively, reaching formation degrees of about 12 and 19% for $Cu(Phen)(H;8,8aPMEA)^+$ and 60 or 73% for Cu(Phen)(8,8aPMEA), respectively.

The calculated stability constants showed no dependence on pH or on the excess of $Cu^{2+}$/Arm employed. The final results for the constants are in each case the averages of the evaluations of five independent pairs of titrations. However, due to the low formation degrees reached for the monoprotonated $M(H;PA)^{\pm}$ species, the stability constants given for these complexes must be considered as estimates. These estimates were further substantiated by comparisons with the known[18] values of the $Cu(Arm)(H;PMEA)^+$ complexes by taking the different acidity constants ($K^H_{H_2(PA)}$) into account.

### *2.3. Spectrophotometric Measurements*

The UV-Vis spectra of the $Cu^{2+}$/Phen/9,8aPMEA or 8,8aPMEA systems were recorded in aqueous solution and 1-cm cells with a Varian Cary 3C spectrophotometer connected to an IBM-compatible desk computer (OS/2 system) and an EPSON stylus 1500 printer. The pH of the solutions was adjusted by dotting with relatively concentrated NaOH and measured with a Metrohm 713 pH meter using a Metrohm 6.204.100 glass electrode. Further details are given in the legend of Figure 4 in Section 3.5.

### 3. RESULTS AND DISCUSSION

All potentiometric pH titrations (25 °C; $I$ = 0.1 M, $NaNO_3$), the results of which are summarized below, were carried out with a ligand concentration of 0.4 mM and at $Cu^{2+}$/Arm concentrations equal to or below 4.4 mM. Under these conditions self-stacking of the ligands is negligibly small as has been shown[18] for PMEA; the same applies to the self-association of $Cu(Phen)^{2+}$.[31] This means, the self-association is negligible for any of the reactants under the present experimental conditions and the results given below certainly refer to monomeric species.

### *3.1. Definition of the Equilibrium Constants*

The ligands 9,8aPMEA$^{2-}$ and 8,8aPMEA$^{2-}$, abbreviated as $PA^{2-}$ (Fig. 1), may bind two protons at the phosphonate group and one at N1 of the adenine moiety. From $H_3(PMEA)^+$ the first proton is released from the $-P(O)(OH)_2$ residue[35] with p$K_a$ = 1.2 and the same may be surmised for the other $H_3(PA)^+$ species. Hence, for the present work only the release of the proton from the $(N1)H^+$ site, followed by the one from the $-P(O)_2(OH)^-$ group need to be considered:

$$H_2(PA)^{\pm} \rightleftharpoons H(PA)^- + H^+ \quad (1a)$$

$$K^H_{H_2(PA)} = [H(PA)^-][H^+]/[H_2(PA)^{\pm}] \quad (1b)$$

$$H(PA)^- \rightleftharpoons PA^{2-} + H^+ \quad (2a)$$

$$K^H_{H(PA)} = [PA^{2-}][H^+]/[H(PA)^-] \quad (2b)$$

Indeed, the experimental data of the potentiometric pH titrations of the $M^{2+}$/PA systems, where $M^{2+} = Cu^{2+}$, $Cu(Bpy)^{2+}$ or $Cu(Phen)^{2+}$, can be fully described by considering the acidity constants of $H_2(PA)^{\pm}$ (eqs (1) and (2)) and the following equilibria (3) and (4),

$$M^{2+} + H(PA)^- \rightleftharpoons M(H;PA)^+ \quad (3a)$$

$$K^M_{M(H;PA)} = [M(H;PA)^+]/([M^{2+}][H(PA)^-]) \quad (3b)$$

$$M^{2+} + PA^{2-} \rightleftharpoons M(PA) \quad (4a)$$

$$K^M_{M(PA)} = [M(PA)]/([M^{2+}][PA^{2-}]) \quad (4b)$$

provided the evaluation of the data is restricted to the pH range below the beginning of the formation of hydroxo complexes which was evident from the titrations of $M^{2+}$ without ligand. It should be noted that in formulas like $M(H;PA)^+$ the $H^+$ and the $PA^{2-}$ are separated by a semicolon to facilitate reading, yet they appear within the same parentheses to indicate that the proton is at the ligand without defining its location.

Equilibria (3a) and (4a) are connected *via* equilibrium (5a), and the corresponding acidity constant (eq. (5b)) may be calculated with equation (6):

$$M(H;PA)^+ \rightleftharpoons M(PA) + H^+ \quad (5a)$$

$$K^H_{M(H;PA)} = [M(PA)][H^+]/[M(H;PA)^+] \quad (5b)$$

$$pK^H_{M(H;PA)} = pK^H_{H(PA)} + \log K^M_{M(H;PA)} - \log K^M_{M(PA)} \quad (6)$$

The equilibrium constants according to equations (3), (4), and (5) are listed in columns 2, 3, and 4 of Table 1, respectively. The acidity constants of the ligands (see footnote "a" in Table 1) and the stability constants of the binary $Cu(H;PA)^+$ and Cu(PA) complexes will be discussed in a different context.[33] Here we concentrate on the properties of the ternary complexes.

**Table 1.** Logarithms of the Stability Constants of the Ternary $Cu(Arm)(H;PA)^+$ (eq. (3)) and Cu(Arm)(PA) (eq. (4)) Complexes as Determined by Potentiometric pH Titrations in Aqueous Solution, Together with the Negative Logarithms of the Acidity Constants (eqs (5) and (6)) of the $Cu(Arm)(H;PA)^+$ Species at 25 °C and $I = 0.1$ M ($NaNO_3$)[a,b]

| M(PA) | $\log K^{M}_{M(H;PA)}$ [c] | $\log K^{M}_{M(PA)}$ | $pK^{H}_{M(H;PA)}$ | $\Delta \log K_{Cu/Arm/PA}$ [d] |
|---|---|---|---|---|
| Cu(9,8aPMEA)[33] | 0.95± 0.25 | 3.98 ± 0.04 | 3.8 ± 0.25 | |
| Cu(Bpy)(9,8aPMEA) | 1.4 ± 0.25 | 4.56 ± 0.06 | 3.7 ± 0.3 | 0.58 ± 0.07 |
| Cu(Phen)(9,8aPMEA) | 1.7 ± 0.25 | 4.81 ± 0.06 | 3.7 ± 0.3 | 0.83 ± 0.07 |
| Cu(8,8aPMEA)[33] | 1.3 ± 0.25 | 3.68 ± 0.06 | 4.4 ± 0.25 | |
| Cu(Bpy)(8,8aPMEA) | 1.7 ± 0.25 | 4.49 ± 0.05 | 4.0 ± 0.3 | 0.81 ± 0.08 |
| Cu(Phen)(8,8aPMEA) | 2.0 ± 0.25 | 4.79 ± 0.07 | 4.0 ± 0.3 | 1.11 ± 0.09 |

[a] The acidity constants of $H_2(9,8aPMEA)^{\pm}$ are $pK^{H}_{H_2(9,8aPMEA)} = 2.73 \pm 0.02$ and $pK^{H}_{H(9,8aPMEA)} = 6.85 \pm 0.02$; those of $H_2(8,8aPMEA)^{\pm}$ are $pK^{H}_{H_2(8,8aPMEA)} = 3.56 \pm 0.02$ and $pK^{H}_{H(8,8aPMEA)} = 6.79 \pm 0.01$.[33],b

[b] The errors given are three times the standard error of the mean value or the sum of the probable systematic errors, whichever is larger. The error limits of the derived data (columns 4 and 5) were calculated according to the error propagation after Gauss.

[c] The values in this column are estimates (see Section 2.2).

[d] Stability constant differences calculated according to eq. (9).

### *3.2. On the Structure of the Monoprotonated Ternary $Cu(Arm)(H;PA)^+$ Complexes*

The analysis of potentiometric pH titrations only yields the amount and distribution of the species of a net charged type; *i.e.*, further information is required to locate the binding sites of the proton and the metal ion in $Cu(Arm)(H;PA)^+$ species. A comparison of the acidity constants of $H_2(9,8aPMEA)^{\pm}$, $pK^{H}_{H_2(9,8aPMEA)} = 2.73$ and $pK^{H}_{H(9,8aPMEA)} = 6.85$, with $pK^{H}_{Cu(Arm)(H;9,8aPMEA)} \approx 3.7$ (Table 1) of the $Cu(Arm)(H;9,8aPMEA)^+$ complexes reveals that the proton in these complexes must be located at the phosphonate group, since metal ion coordination must give rise to an acidification,[36,37] which amounts to $\Delta\, pK_a = pK^{H}_{H(9,8aPMEA)} - pK^{H}_{Cu(Arm)(H;9,8aPMEA)} = (6.85 \pm 0.02) - (3.7 \pm 0.3) = 3.15 \pm 0.3$ in the present case. In the $Cu(Arm)(H;8,8aPMEA)^+$ species the proton is also clearly bound at the phosphonate group though the acidification due to the metal ion might be somewhat less pronounced: $\Delta\, pK_a = pK^{H}_{H(8,8aPMEA)} - pK^{H}_{Cu(Arm)(H;8,8aPMEA)} = (6.79 \pm 0.01) - (4.0 \pm 0.3) = 2.8 \pm 0.3$.

Where is the $Cu(Arm)^{2+}$ unit located? In principle, there are two possibilities: One, where $Cu(Arm)^{2+}$ is stacked with the purine system of $H(PA)^-$, designated as $[Cu(Arm)/(H;PA)]^+_{st}$, and another one, where $Cu(Arm)^{2+}$ is coordinated either to the N1/N7 sites of the adenine residue (see [24]), $[H;PA{\cdot}Cu(Arm)]^+_{ade}$, or to the phosphonate group which already carries the proton. However, the formation of this latter species with both the proton and $Cu(Arm)^{2+}$ at the phosphonate group is unlikely, in agreement with previous conclusions.[24] Hence, we are left with the species $[H;PA{\cdot}Cu(Arm)]^+_{ade}$ and $[Cu(Arm)/(H;PA)]^+_{st}$ and we have to consider the intramolecular equilibrium (7):

$$[H;PA{\cdot}Cu(Arm)]^+_{ade} \rightleftharpoons [Cu(Arm)/(H;PA)]^+_{st} \tag{7}$$

An evaluation following exactly the route described in [2] leads to the conclusion that for all four $Cu(Arm)(H;PA)^+$ systems the stacked species in equilibrium (7) dominate with formation degrees of more than 70%, most likely being between 80 and 95%. As one might expect, the formation degree of $[Cu(Phen)/(H;PA)]^+_{st}$ is about 10% larger than the one of $[Cu(Bpy)/(H;PA)]^+_{st}$.

### *3.3. Proof of an Increased Stability of the Mixed Ligand Cu(Arm)(PA) Complexes*

The stability of mixed-ligand complexes[38,39] may be quantified by considering equilibrium (8a); the corresponding equilibrium constant is calculated with equation (9).

$$Cu(Arm)^{2+} + Cu(PA) \rightleftharpoons Cu(Arm)(PA) + Cu^{2+} \tag{8a}$$

$$10^{\Delta \log K_{Cu/Arm/PA}} = \frac{[Cu(Arm)(PA)]\,[Cu^{2+}]}{[Cu(Arm)^{2+}]\,[Cu(PA)]} \tag{8b}$$

$$\Delta \log K_{Cu/Arm/PA} = \log K^{Cu(Arm)}_{Cu(Arm)(PA)} - \log K^{Cu}_{Cu(PA)} \tag{9}$$

According to the general rule for complex stabilities, $K_1 > K_2$, equilibrium (8a) is expected to be on the left side with negative values for $\Delta \log K_{Cu/Arm/PA}$, in agreement with statistical considerations,[38,39] *i.e.*, $\Delta \log K_{Cu/statist} \approx -0.5$.[39] From the values listed in column 5 of Table 1 it is evident that equilibrium (8) is significantly displaced to the right side. More important, however, is a comparison with the results obtained for the dianion of phosphonomethoxyethane ($PME^{2-}$), $CH_3CH_2$-O-$CH_2$-$PO_3^{2-}$, which reflects the properties of the side chain of 9,8aPMEA$^{2-}$ and 8,8aPMEA$^{2-}$ (Fig. 1). Indeed, these results, $\Delta \log K_{Cu/Bpy/PME} = 0.13 \pm 0.04$ and $\Delta \log K_{Cu/Phen/PME} = 0.17 \pm 0.05$,[31] are considerably smaller than the values of $\Delta \log K_{Cu/Arm/PA}$ for the Cu(Arm)(PA) complexes, which means that the adenine residue contributes to the stability of the Cu(Arm)(PA) species. This comparison thus provides the first clear hint for the occurrence of an intramolecular stacking interaction in these latter mentioned complexes.

Another way to evaluate the increased stability of ternary $Cu^{2+}$ complexes, independently of the properties of the binary Cu(9,8aPMEA) and Cu(8,8aPMEA) species, rests on the previously established[20,31] straight-line correlations for $\log K^{Cu(Arm)}_{Cu(Arm)(R\text{-}PO_3)}$ *versus* $pK^{H}_{H(R\text{-}PO_3)}$ plots (eqs (10) and (11)), where R-$PO_3^{2-}$ represents phosphate monoester or phosphonate ligands in which the residue R is unable to interact with $Cu(Arm)^{2+}$:

$$\log K^{Cu(Bpy)}_{Cu(Bpy)(R\text{-}PO_3)} = 0.465 \times pK^{H}_{H(R\text{-}PO_3)} + 0.009 \quad (10)$$

$$\log K^{Cu(Phen)}_{Cu(Phen)(R\text{-}PO_3)} = 0.465 \times pK^{H}_{H(R\text{-}PO_3)} + 0.018 \quad (11)$$

The error limits of log stability constants calculated with given $pK^{H}_{H(R\text{-}PO_3)}$ values and equations (10) and (11) are ±0.07 and ±0.06 (3σ) log units, respectively, in the pH range 5-8.[31]

The reference lines as defined by equations (10) and (11) are shown in Figure 2, where the stability constants $\log K^{Cu(Arm)}_{Cu(Arm)(PA)}$ *versus* the acidity constants $pK^{H}_{H(PA)}$ of the 9,8aPMEA and 8,8aPMEA species are also plotted, together with the corresponding data[31] of the Cu(Arm)(PME) systems. All these data points are above their reference lines, proving an increased complex stability which must mean[40] that aside from the $Cu(Arm)^{2+}$-phosphonate coordination further interactions take place. The vertical distances in Figure 2 between the data points due to Cu(Arm)(9,8aPMEA), Cu(Arm)(8,8aPMEA) and Cu(Arm)(PME) and the reference lines are a measure for the extent of the intramolecular interactions in these complexes and they can be defined according to equation (12) (in this case, $PA^{2-}$ also represents $PME^{2-}$):

$$\log \Delta_{Cu/Arm/PA} = \log K^{Cu(Arm)}_{Cu(Arm)(PA)} - \log K^{Cu(Arm)}_{Cu(Arm)(PA)op} \quad (12a)$$

$$= \log K^{Cu(Arm)}_{Cu(Arm)(PA)exptl} - \log K^{Cu(Arm)}_{Cu(Arm)(PA)calcd} \quad (12b)$$

The expressions $\log K^{Cu(Arm)}_{Cu(Arm)(PA)calcd}$ (eq. (12b)) and $\log K^{Cu(Arm)}_{Cu(Arm)(PA)op}$ (eq. (12a)) are synonymous because the calculated value equals the stability constant of the 'open' isomer, $Cu(Arm)(PA)_{op}$, in which only a -$PO_3^{2-}$/$Cu(Arm)^{2+}$ interaction occurs. The first term on the right hand side in equation (12) is the experimentally determined stability constant (eq. (4)), whereas a value for $\log K^{Cu(Arm)}_{Cu(Arm)(PA)calcd}$ can be calculated with the acidity constant $pK^{H}_{H(PA)}$ and the straight-line equations (10) and (11). As indicated above, such a calculated value quantifies the stability of the open isomer.

The ligand $PME^{2-}$ offers to metal ions the phosphonate group for coordination, but an interaction with the ether oxygen is also possible as has repeatedly been proven.[18-22,41,42] This gives rise to 5-membered chelates and therefore equilibrium (13a) needs to be considered:

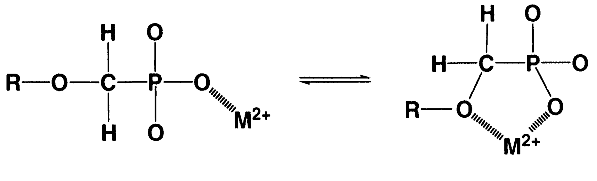

(13a)

$$K_I = [Cu(Arm)(PME)_{cl}]/[Cu(Arm)(PME)_{op}] \quad (13b)$$

The dimensionless equilibrium constant $K_I$ (eq. (13b)) is calculated according to equation (14),

$$K_I = 10^{\log \Delta_{Cu/Arm/PME}} - 1 \quad (14)$$

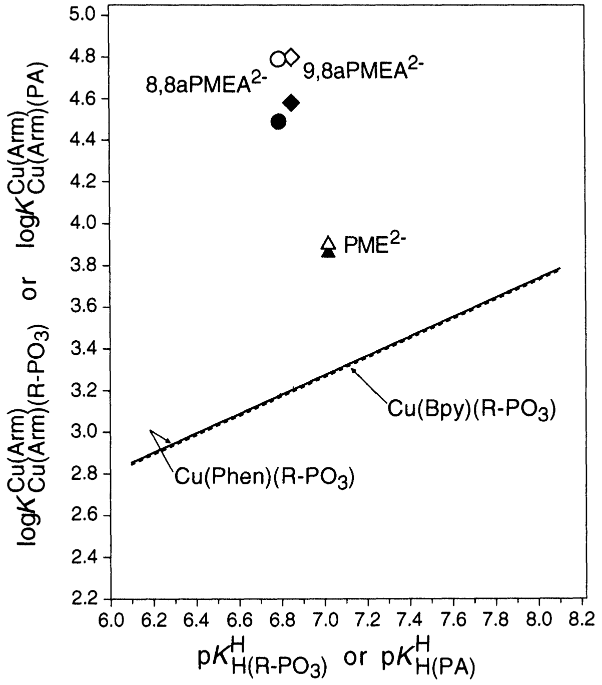

**Figure 2.** Evidence for an enhanced stability of the ternary Cu(Arm)(9,8aPMEA) (◇,◆), Cu(Arm)(8,8aPMEA) (○,●), and Cu(Arm)(PME) (△,▲) complexes based on the relationship between log $K^{Cu(Arm)}_{Cu(Arm)(R\text{-}PO_3)}$ or log $K^{Cu(Arm)}_{Cu(Arm)(PA)}$ and $pK^H_{H(R\text{-}PO_3)}$ or $pK^H_{H(PA)}$ in aqueous solution at $I$ = 0.1 M ($NaNO_3$) and 25 °C. The plotted data for 9,8aPMEA and 8,8aPMEA are from Table 1 and those for PME from [31]. The two reference lines represent the log $K^{Cu(Arm)}_{Cu(Arm)(R\text{-}PO_3)}$ *versus* $pK^H_{H(R\text{-}PO_3)}$ relationship for ternary Cu(Arm)(R-$PO_3$) complexes (eqs (10) and (11)); R-$PO_3^{2-}$ symbolizes phosphonates or phosphate monoesters in which the group R is unable to undergo any kind of hydrophobic, stacking or other type of interactions, *i.e.* ligands like D-ribose 5-monophosphate, methanephosphonate or ethanephosphonate.[31] The broken line holds for Arm = Bpy and the solid line for Arm = Phen. Both straight lines represent the situation for ternary complexes without an intramolecular ligand-ligand interaction. The vertical dotted lines emphasize the stability differences from the reference lines; they equal log $\Delta_{Cu/Arm/PA}$ as defined in equation (12).

and now, knowing $K_I$, the percentage of the closed isomer, $Cu(Arm)(PME)_{cl}$, in equilibrium (13a) can be obtained with equation (15):

$$\% \, Cu(Arm)(PME)_{cl} = 100 \cdot K_I/(1 + K_I) \quad (15)$$

**Table 2.** Quantification of the Stability Increase *via* log $\Delta_{Cu/Arm/PA}$ (eq. (12)) for the Cu(Arm)(PA) Complexes, where PA = 9,8aPMEA$^{2-}$, 8,8aPMEA$^{2-}$ or PME$^{2-}$, and Arm = Bpy or Phen, Together with the Extent of the Intramolecular Chelate Formation (eq. (13)) in the Cu(Arm)(PME) Species[31] in Aqueous Solution at 25 °C and $I$ = 0.1 M ($NaNO_3$)[a]

| Cu(Arm)(PA) | log $K^{Cu(Arm)}_{Cu(Arm)(PA)exptl}$ [b] | log $K^{Cu(Arm)}_{Cu(Arm)(PA)calcd}$ [c] | log $\Delta_{Cu/Arm/PA}$ | $K_I$ [d] | %Cu(Arm)(PME)$_{cl}$ [e] |
|---|---|---|---|---|---|
| Cu(Bpy)(9,8aPMEA) | 4.56 ± 0.06 | 3.19 ± 0.07 | 1.37 ± 0.09 | | |
| Cu(Phen)(9,8aPMEA) | 4.81 ± 0.06 | 3.20 ± 0.06 | 1.61 ± 0.08 | | |
| Cu(Bpy)(8,8aPMEA) | 4.49 ± 0.05 | 3.17 ± 0.07 | 1.32 ± 0.09 | | |
| Cu(Phen)(8,8aPMEA) | 4.79 ± 0.07 | 3.18 ± 0.06 | 1.61 ± 0.09 | | |
| Cu(Bpy)(PME)[31] | 3.86 ± 0.03 | 3.27 ± 0.07 | 0.59 ± 0.08 | 2.89 ± 0.68 | 74 ± 5 |
| Cu(Phen)(PME)[31] | 3.90 ± 0.04 | 3.28 ± 0.06 | 0.62 ± 0.07 | 3.17 ± 0.69 | 76 ± 4 |

[a] For the error limits see footnote 'b' of Table 1.

[b] These values are from column 3 in Table 1.

[c] These constants were calculated with eqs (10) or (11) and the $pK^H_{H(PA)}$ values given in footnote 'a' of Table 1.

[d] See eqs (13b) and (14).

[e] Calculated according to equation (15).

From the results given in the lower part of Table 2 it is evident that the closed isomer of Cu(Arm)(PME) is an important species with a formation degree of about 75%. Naturally, the formation of the corresponding isomer involving the ether oxygen is also to be expected (see Fig. 1) for the Cu(Arm)(9,8aPMEA) and for Cu(Arm)(8,8aPMEA) systems and we designate it as Cu(Arm)(PA)$_{cl/O}$. However, the log $\Delta_{Cu/Arm/PA}$ values listed in column 4 of Table 2 are by about 0.7 to 1 log unit larger for the latter mentioned complexes than for the Cu(Arm)(PME) species and this must mean that in the systems with 9,8aPMEA and 8,8aPMEA, next to Cu(Arm)(PA)$_{op}$ and Cu(Arm)(PA)$_{cl/O}$, a third isomer must occur which involves the adenine residue. The ligands 9,8aPMEA$^{2-}$ and 8,8aPMEA$^{2-}$ offer only two such possibilities: The phosphonate-coordinated Cu(Arm)$^{2+}$ forms (i) a macrochelate with one of the nitrogens of the adenine residue, or (ii) an intramolecular stack between the aromatic ring systems of Bpy/Phen and the adenine moiety. That the first possibility is not of relevance has been discussed in detail for 3'-deoxa-PMEA,[2] and the same arguments also apply here, whereas for the second possibility involving intramolecular stacks, many examples exist.[2,28,30,31,39,40,43] Hence, the additional enhanced complex stability may be attributed indeed to intramolecular stack formation.

Application of space-filling molecular models reveals that the adenine residue of the 9,8aPMEA or 8,8aPMEA ligands, which are equatorially chelated to Cu(Arm)$^{2+}$ *via* the phosphonate group and the ether oxygen, cannot stack well with the aromatic rings of the also equatorially coordinated Arm; a substantial and strain-free overlap of the aromatic systems is only possible if the ether oxygen is not equatorially coordinated to $Cu^{2+}$. This latter situation is depicted in Figure 3 for 9,8aPMEA. However, from the molecular models it is also evident that an *apical* ether oxygen coordination and simultaneous stack formation would be compatible with each other in the Cu(Arm)(PA) species with PA$^{2-}$ = 9,8aPMEA or 8,8aPMEA. Hence, there are various intramolecularly stacked Cu(Arm)(PA) species possible including those with somewhat different orientations of the aromatic rings toward each other. As there is at present no way to distinguish these various isomers and conformers from each other, we treat all the stacked species together and designate them as Cu(Arm)(PA)$_{st}$. The sum of the above reasonings then gives rise to the equilibrium scheme (16), where the pure phosphonate-coordinated isomer is designated as Cu(Arm)(PA)$_{op}$. It is evident that the upper branch of this equilibrium scheme reflects equilibrium (13a) while the lower branch reflects the stacking interaction (Fig. 3).

$$\mathrm{Cu(Arm)^{2+} + PA^{2-} \rightleftharpoons Cu(Arm)(PA)_{op}} \quad \begin{cases} \xrightarrow{K_{I/O}} \mathrm{Cu(Arm)(PA)_{cl/O}} \\ \xrightarrow{K_{I/st}} \mathrm{Cu(Arm)(PA)_{st}} \end{cases} \qquad (16)$$

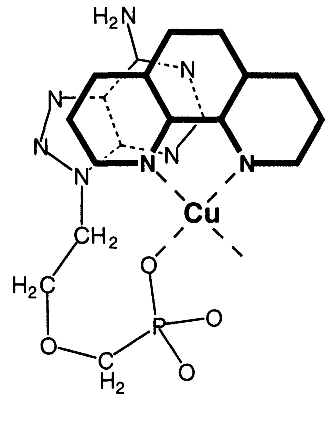

**Figure 3.** Tentative and simplified structure of a Cu(Phen)(9,8aPMEA) species with an intramolecular stack. The orientation of the aromatic rings may vary among the stacked species; such a stacked complex in solution should not be considered as being rigid.

### *3.4. Evaluation of the Intramolecular Equilibria Involving Three Different Cu(Arm)(PA) Species*

Based on the equilibrium scheme (16) the corresponding equilibrium constants can be defined as given in equations (17)-(19):

$$\log K^{Cu(Arm)}_{Cu(Arm)(PA)op} = [Cu(Arm)(PA)_{op}]/([Cu(Arm)^{2+}]\,[PA^{2-}]) \quad (17)$$

$$K_{I/O} = [Cu(Arm)(PA)_{cl/O}]/[Cu(Arm)(PA)_{op}] \quad (18)$$

$$K_{I/st} = [Cu(Arm)(PA)_{st}]/[Cu(Arm)(PA)_{op}] \quad (19)$$

With these definitions the experimentally accessible equilibrium constant (4b) can be reformulated as equation (20):[20a,31]

$$K^{Cu(Arm)}_{Cu(Arm)(PA)} = \frac{[Cu(Arm)(PA)]}{[Cu(Arm)^{2+}]\,[PA^{2-}]} \quad (4b)$$

$$= \frac{([Cu(Arm)(PA)_{op}] + [Cu(Arm)(PA)_{cl/O}] + [Cu(Arm)(PA)_{st}])}{[Cu(Arm)^{2+}]\,[PA^{2-}]} \quad (20a)$$

$$= K^{Cu(Arm)}_{Cu(Arm)(PA)op} + K_{I/O} \cdot K^{Cu(Arm)}_{Cu(Arm)(PA)op} + K_{I/st} \cdot K^{Cu(Arm)}_{Cu(Arm)(PA)op} \quad (20b)$$

$$= K^{Cu(Arm)}_{Cu(Arm)(PA)op}\,(1 + K_{I/O} + K_{I/st}) \quad (20c)$$

From here one arrives easily[31] at equation (21), where $Cu(Arm)(PA)_{int/tot}$ refers to the sum of all the species with an intramolecular interaction:

$$K_I^* = K_{I/tot} = \frac{K^{Cu(Arm)}_{Cu(Arm)(PA)}}{K^{Cu(Arm)}_{Cu(Arm)(PA)_{op}}} - 1 = 10^{\log \Delta_{Cu/Arm/PA}} - 1 \quad (21a)$$

$$K_I^* = K_{I/tot} = \frac{[Cu(Arm)(PA)_{int/tot}]}{[Cu(Arm)(PA)_{op}]} \quad (21b)$$

$$= \frac{[Cu(Arm)(PA)_{cl/O}] + [Cu(Arm)(PA)_{st}]}{[Cu(Arm)(PA)_{op}]} \quad (21c)$$

$$= K_{I/O} + K_{I/st} \quad (21d)$$

In those instances where the stacked species do not form, the above equations reduce to the two-isomer problem treated in equations (13) and (14). It is evident that $K_I^* = K_{I/tot}$ according to equation (21a) can be calculated *via* the values log $\Delta_{Cu/Arm/PA}$ as defined by equation (12) and listed in the upper part of column 4 in Table 2.

**Table 3.** Intramolecular Equilibrium Constants for the Formation of the Three Differently Structured Cu(Arm)(PA) Species Shown in the Equilibrium Scheme (16), Together with the Percentages in Which These Species Occur in Aqueous Solution at 25 °C and $I = 0.1$ M ($NaNO_3$)[a]

| No. | Cu(Arm)(PA) | log $\Delta_{Cu/Arm/PA}$ | $K_I^* = K_{I/tot}$ | %$Cu(Arm)(PA)_{int/tot}$ | %$Cu(Arm)(PA)_{op}$ |
|---|---|---|---|---|---|
| 1a | Cu(Bpy)(9,8aPMEA) | 1.37 ± 0.09 | 22.44 ± 4.86 | 95.73 ± 0.88 | 4.27 ± 0.88 |
| 2a | Cu(Phen)(9,8aPMEA) | 1.61 ± 0.08 | 39.74 ± 7.50 | 97.55 ± 0.45 | 2.45 ± 0.45 |
| 3a | Cu(Bpy)(8,8aPMEA) | 1.32 ± 0.09 | 19.89 ± 4.33 | 95.21 ± 0.99 | 4.79 ± 0.99 |
| 4a | Cu(Phen)(8,8aPMEA) | 1.61 ± 0.09 | 39.74 ± 8.44 | 97.55 ± 0.51 | 2.45 ± 0.51 |
| No. | Cu(Arm)(PA) | $K_{I/O}$ | $K_{I/st}$ | %$Cu(Arm)(PA)_{cl/O}$[b] | %$Cu(Arm)(PA)_{st}$[c] |
| 1b | Cu(Bpy)(9,8aPMEA) | 2.89 ± 0.68 | 19.55 ± 4.91 | 12.3 ± 3.9 | 83.4 ± 4.0 |
| 2b | Cu(Phen)(9,8aPMEA) | 3.17 ± 0.69 | 36.57 ± 7.53 | 7.8 ± 2.2 | 89.8 ± 2.2 |
| 3b | Cu(Bpy)(8,8aPMEA) | 2.89 ± 0.68 | 17.00 ± 4.38 | 13.8 ± 4.3 | 81.4 ± 4.4 |
| 4b | Cu(Phen)(8,8aPMEA) | 3.17 ± 0.69 | 36.57 ± 8.47 | 7.8 ± 2.3 | 89.8 ± 2.4 |

[a] The values listed in the third column of the upper part are from the fourth column in the upper part of Table 2. The values for $K_I^* = K_{I/tot}$ follow from eq. (21a) and %$Cu(Arm)(PA)_{int/tot}$ is calculated analogously to eq. (15). The values given in the sixth column for %$Cu(Arm)(PA)_{op}$ follow from 100 – %$Cu(Arm)(PA)_{int/tot}$. The constants for $K_{I/O}$ in column 3 of the lower part are from column 5 in the lower part of Table 2 (for the corresponding justification[31] see also text in Section 3.4); with eq. (21d) and the now known values for $K_I^*$ and $K_{I/O}$ that for $K_{I/st}$ may be calculated (column 4 in the lower part). All error limits correspond to three times the standard deviation (3σ); they were calculated according to the error propagation after Gauss.

[b] These values were calculated *via* eq. (18) with $K_{I/O}$ and %$Cu(Arm)(PA)_{op}$.

[c] The values for %$Cu(Arm)(PA)_{st}$ follow from the difference %$Cu(Arm)(PA)_{int/tot}$ – %$Cu(Arm)(PA)_{cl/O}$ (cf. eqs (21b) and (21c)); %$Cu(Arm)(PA)_{st}$ may also be calculated *via* eq. (19) with $K_{I/st}$ and %$Cu(Arm)(PA)_{op}$. The results are the same for both calculation methods yet the error limits are understandably larger for the second method (data not shown).

The resulting $K_I^*$ values are given in the fourth column of the upper part of Table 3 and they allow to calculate the concentrations of the open isomers, $Cu(Arm)(PA)_{op}$. To be able to calculate the formation degree of the species that form the five-membered chelate with the ether oxygen, *i.e.* $Cu(Arm)(PA)_{cl/O}$ (eq. (18)), we made the justified assumption that $Cu(Arm)(PME)_{cl}$ (Section 3.3; Table 2)[31] and $Cu(Arm)(PA)_{cl/O}$ have the same stability, *i.e.* that the equilibrium constant $K_{I/O}$ for $Cu(Arm)(PA)_{cl/O}$ equals the corresponding value (= $K_I$) for $Cu(Arm)(PME)_{cl}$. Knowledge of $K_I^*$ and $K_{I/O}$ permits now to calculate $K_{I/st}$ by using equation (21d) and hence the formation degree of the $Cu(Arm)(PA)_{st}$ species. Finally, the difference between 100 and the sum of the percentages for $Cu(Arm)(PA)_{op}$ and $Cu(Arm)(PA)_{cl/O}$ will, of course, also result in % $Cu(Arm)(PA)_{st}$ and $K_{I/st}$. The results of these calculations are summarized in the lower part of Table 3.

Considering the equilibrium scheme (16) and the corresponding results summarized in Table 3 several conclusions are evident: (i) All three structurally different species are formed in appreciable amounts in the Cu(Phen)(9,8aPMEA) and Cu(Phen)(8,8aPMEA) systems. (ii) The stacked species (Fig. 3) clearly dominate, reaching formation degrees of about 80 to 90%. (iii) Consequently, the formation degree of the five-membered chelates involving the ether oxygen is suppressed, roughly speaking to about 10%, compared with the approximately 75% present in the Cu(Arm)(PME) systems (*cf.* Table 2).

A further aspect that warrants emphasis is the fact that the values for $K_{I/st}$ are lower by a factor of about one half for the complexes containing Bpy compared with those containing Phen (Table 3, column 4 in the lower part). The corresponding observation has previously been made in studies with adenosine 5'-monophosphate ($AMP^{2-}$)[44] and other adenine derivatives.[31] Evidently this is the result of the smaller aromatic-ring system of 2,2'-bipyridine, compared to that of 1,10-phenanthroline, which gives rise to a less pronounced overlap with the adenine residue.

### *3.5. Spectrophotometric Confirmation of Stacking in the Cu(Phen)(PA) Systems*

The results of Section 3.4 provide very clear though indirect evidence, obtained *via* stability constant comparisons, for stack formation in the ternary complexes. Direct evidence for the formation of the stacks can be obtained *via* either $^1H$ NMR shift experiments[28,45] or spectrophotometric measurements.[28,30,46,47] The first-mentioned method is excluded because of the line broadening effects of $Cu^{2+}$. However, the second method, which is based on the experience that the formation of stacked adducts is connected with the observation of charge-transfer bands,[28,30,46,47] should be suitable.

Indeed, the spectrophotometric measurements carried out for the Cu(Phen)(9,8aPMEA) and Cu(Phen)(8,8aPMEA) systems, which are summarized in Figure 4, confirm the formation of stacks in the ternary species. The difference spectra reveal the occurrence of new absorption bands at approximately 335 and 350 nm in accordance with previous observations of various $Cu(Bpy)^{2+}$ [46] and $Cu(Phen)^{2+}$ [47] systems with nucleotides.[30,48]

The spectrophotometric measurements seen in Figure 4 are not very precise. It was difficult to adjust a pH of 4.5 in the solutions without obtaining a precipitation of hydroxo complexes and the values of the difference-absorptions ($\Delta$A) measured are very small. Therefore, a quantitative evaluation of these data is not appropriate; important for the present context is the simple fact that the data confirm stacking.

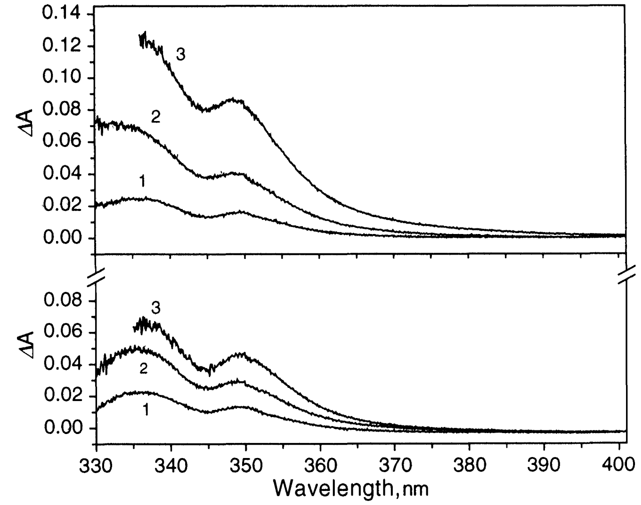

**Figure 4.** Difference absorption spectra for the ternary $Cu^{2+}$/Phen/9,8aPMEA (lower part) and $Cu^{2+}$/Phen/8,8aPMEA (upper part) systems measured in 1-cm cells, *i.e.*, the reference beam contained one cell with $Cu^{2+}$/Phen and a second one with the corresponding azaPMEA derivative; the sample beam contained one cell with the mixed ligand system and one with water. The azaPMEA concentrations were $10^{-3}$ M and those of $Cu(NO_3)_2$/Phen were $1.32 \times 10^{-3}$ M (1), $2.64 \times 10^{-3}$ M (2) and $5.28 \times 10^{-3}$ M (3) [$Cu(Phen)^{2+}$ is nearly completely formed under the given conditions]. $NaNO_3$ was added to all four solutions to maintain $I$ = 0.1 M. The pH was always adjusted to 4.50 ± 0.02 by dotting with relatively concentrated NaOH (at higher pH hydroxo-complex formation occurs). See also Section 2.3.

### 4. CONCLUSIONS

The present study reveals several interesting properties of PMEA and its derivatives: (i) It is relatively astonishing to observe that the binding of the side chain either at N9 or at N8 (see Fig. 1) does not affect the stacking properties in the ternary complexes studied; *i.e.*, the extent of stacking is within the error limits identical in Cu(Arm)(9,8aPMEA) and Cu(Arm)(8,8aPMEA) (Table 3, column 6 in the lower part). This means most likely that the side chain is long enough to provide the flexibility needed for a favorable orientation of the aromatic rings in the stacks in both types of complexes. (ii) The extent of stacking of the two azaPMEAs in their $Cu(Arm)^{2+}$ complexes is within the error limits identical with that of PMEA[31] and 3'-deoxa-PMEA[2] in the corresponding ternary complexes. This means, the deletion of the ether oxygen from the side chain as well as the presence of an 'extra' nitrogen atom in the adenine residue do not affect the stacking properties of the purine system. (iii) Furthermore, even the stacking properties of the Cu(Arm)(AMP) species[44] are within the error limits identical with those of the mentioned ternary complexes. In other words, as far as the stacking properties are concerned, all of the mentioned PMEA derivatives resemble closely the parent nucleotide, adenosine 5'-monophosphate ($AMP^{2-}$).

A further point of interest is that 9,8aPMEA shows *in vitro* some antiviral activity whereas the 8,8a isomer does not.[49,50] Why? Based on the presented results one may suggest that both nucleotide analogues could possibly bind *via* stacking to the active site of the enzyme in question, but that the orientation within the active site is different preventing thus the desired[26] biological action.

### ACKNOWLEDGEMENTS

The competent technical assistance of Mrs. Rita Baumbusch and Mrs. Astrid Sigel in the preparation of this manuscript is gratefully acknowledged. This study was supported by the Swiss National Science Foundation (H.S.) and the Grant Agency of the Czech Republic (203/96/K001; A.H.) as well as within the COST D8 and D20 programmes by the Swiss Federal Office for Education and Science (H.S.) and the Ministry of Education of the Czech Republic (A.H.).

### REFERENCES

1. This is part 66 of the series 'Ternary Complexes in Solution'; for part 65 see [2] and for part 64 see: Da Costa, C. P.; Song, B.; Gregáň, F.; Sigel, H.: *J. Chem. Soc., Dalton Trans.* (2000) 899-904.
2. Gómez-Coca, R. B.; Kapinos, L. E.; Holý, A.; Vilaplana, R. A.; González-Vílchez, F.; Sigel, H.: *J. Inorg. Biochem.* (2001) in press.
3. Martin, J. C.; (ed.): *Nucleotide Analogues as Antiviral Agents*; *ACS Symposium Series* (1989) **401**, American Chemical Society, Washington, DC.
4. Holý, A.; Rosenberg, I.: *Coll. Czech. Chem. Commun.* (1987) **52**, 2801-2809.
5. De Clercq, E.; Sakuma, T.; Baba, M.; Pauwels, R.; Balzarini, J.; Rosenberg, I.; Holý, A.: *Antiviral Res.* (1987) **8**, 261-272.
6. De Clercq, E.: *Coll. Czech. Chem. Commun.* (1998) **63**, 449-479 and 480-506.
7. De Clercq, E.: *Pure Appl. Chem.* (1998) **70**, 567-577.
8. Colledge, D.; Civitico, G.; Locarnini, S.; Shaw, T.: *Antimicrob. Agents Chemother.* (2000) **44**, 551-560.
9. Franěk, F.; Holý, A.; Votruba, I.; Eckschlager, T.: *Internat. J. Oncol.* (1999) **14**, 745-752.
10. Hatse, S.; Naesens, L.; Degrève, B.; Segers, C.; Vandeputte, M.; Waer, M.; De Clercq, E.; Balzarini, J.: *Int. J. Cancer* (1998) **76**, 595-600.
11. Zídek, Z.; Franková, D.; Holý, A.: *Eur. J. Pharmacol.* (1999) **376**, 91-100.
12. Holý, A.; Günter, J.; Dvořáková, H.; Masojídková, M.; Andrei, G.; Snoeck, R.; Balzarini, J.; De Clercq, E.: *J. Med. Chem.* (1999) **42**, 2064-2086.
13. Balzarini, J.; Hao, Z.; Herdewijn, P.; Johns, D. G.; De Clercq, E.: *Proc. Natl. Acad. Sci. USA* (1991) **88**, 1499-1503.
14. Birkuš, G.; Votruba, I.; Holý, A.; Otová, B.: *Biochem. Pharmacol.* (1999) **58**, 487-492.
15. Sigel, H.; Sigel, A.; (eds): *Interrelations among Metal Ions, Enzymes, and Gene Expression*, Vol. 25 of *Met. Ions Biol. Syst.*; M. Dekker, Inc.; New York, Basel; 1989, pp 1-557.
16. Crowley, J. D.; Traynor, D. A.; Weatherburn, D. C.: *Met. Ions Biol. Syst.* (2000) **37** (*Manganese and Its Role in Biological Processes*), 209-278.
17. Mildvan, A. S.: *Magnesium* (1987) **6**, 28-33.
18. Sigel, H.; Chen, D.; Corfù, N. A.; Gregáň, F.; Holý, A.; Strašák, M.: *Helv. Chim. Acta* (1992) **75**, 2634-2656.
19. Chen, D.; Gregáň, F.; Holý, A.; Sigel, H.: *Inorg. Chem.* (1993) **32**, 5377-5384.
20. (a) Sigel, H.: *Coord. Chem. Rev.* (1995) **144**, 287-319. (b) Sigel, H.: *J. Indian Chem. Soc.* (1997) **74**, 261-271 (P. Ray Award Lecture).
21. Sigel, H.; Song, B.: *Met. Ions Biol. Syst.* (1996) **32** (*Interactions of Metal Ions with Nucleotides, Nucleic Acids, and Their Constituents*), 135-205.
22. Blindauer, C. A.; Emwas, A. H.; Holý, A.; Dvořáková, H.; Sletten, E.; Sigel, H.: *Chem. Eur. J.* (1997) **3**, 1526-1536.
23. Sigel, H.; Blindauer, C. A.; Holý, A.; Dvořáková, H.: *Chem. Commun.* (1998) 1219-1220.
24. Gómez-Coca, R. B.; Kapinos, L. E.; Holý, A.; Vilaplana, R. A.; González-Vílchez, F.; Sigel, H.: *J. Chem. Soc., Dalton Trans.* (2000) 2077-2084.
25. Sigel, H.; Song, B.; Blindauer, C. A.; Kapinos, L. E.; Gregáň, F.; Prónayová, N.: *Chem. Commun.* (1999) 743-744.

26. Sigel, H.: *Pure Appl. Chem.* (1999) **71**, 1727-1740.
27. Sigel, H.: *Pure Appl. Chem.* (1998) **70**, 969-976.
28. Yamauchi, O.; Odani, A.; Masuda, H.; Sigel, H.: *Met. Ions Biol. Syst.* (1996) **32** (*Interactions of Metal Ions with Nucleotides, Nucleic Acids, and Their Constituents*), 207-270.
29. (a) Fischer, B. E.; Sigel, H.: *J. Am. Chem. Soc.* (1980) **102**, 2998-3008. (b) Sigel, H.; Tribolet, R.; Scheller, K. H.: *Inorg. Chim. Acta* (1985) **100**, 151-164.
30. Lüth, M. S.; Song, B.; Lippert, B.; Sigel, H.: *Inorg. Chem.* (2000) **39**, 1305-1310.
31. Chen, D.; Bastian, M.; Gregáň, F.; Holý, A.; Sigel, H.: *J. Chem. Soc., Dalton Trans.* (1993) 1537-1546.
32. Holý, A.; Dvořáková, H.; Jindřich, J.; Masojídková, M.; Buděšínský, M.; Balzarini, J.; Andrei, G.; De Clercq, E.: *J. Med. Chem.* (1996) **39**, 4073-4088.
33. Gómez-Coca, R. B.; Kapinos, L. E.; Holý, A.; Vilaplana, R. A.; González-Vílchez, F.; Sigel, H.: details on the binary complexes to be published.
34. (a) Irving, H.; Mellor, D. H.: *J. Chem. Soc.* (1962) 5222-5237. (b) Anderegg, G.: *Helv. Chim. Acta* (1963) **46**, 2397-2410.
35. Blindauer, C. A.; Holý, A.; Dvořáková, H.; Sigel, H.: *J. Chem. Soc., Perkin Trans. 2* (1997) 2353-2363.
36. Sigel, H.; Lippert, B.: *Pure Appl. Chem.* (1998) **70**, 845-854.
37. Song, B.; Zhao, J.; Griesser, R.; Meiser, C; Sigel, H.; Lippert, B.: *Chem. Eur. J.* (1999) **5**, 2374-2387.
38. Sigel, H.: *Angew. Chem., Int. Ed. Engl.* (1975) **14**, 394-402.
39. Malini-Balakrishnan, R.; Scheller, K. H.; Häring, U. K.; Tribolet, R.; Sigel, H.: *Inorg. Chem.* (1985) **24**, 2067-2076.
40. Martin, R. B.; Sigel, H.: *Comments Inorg. Chem.* (1988) **6**, 285-314.
41. Blindauer, C. A.; Holý, A.; Sigel, H.: *Coll. Czech. Chem. Commun.* (1999) **64**, 613-632.
42. Blindauer, C. A.; Sjåstad, T. I.; Holý, A.; Sletten, E.; Sigel, H.: *J. Chem. Soc., Dalton Trans.* (1999) 3661-3671.
43. Sigel, H.: *Pure Appl. Chem.* (1989) **61**, 923-932.
44. Massoud, S. S.; Tribolet, R.; Sigel, H.: *Eur. J. Biochem.* (1990) **187**, 387-393.
45. (a) Sigel, H.; Malini-Balakrishnan, R.; Häring, U. K.: *J. Am. Chem. Soc.* (1985) **107**, 5137-5148. (b) Liang, G.; Tribolet, R.; Sigel, H.: *Inorg. Chem.* (1988) **27**, 2877-2887.
46. (a) Naumann, C. F.; Sigel, H.: *J. Am. Chem. Soc.* (1974) **96**, 2750-2756. (b) Chaudhuri, P.; Sigel, H.: *J. Am. Chem. Soc.* (1977) **99**, 3142-3150. (c) Farkas, E.; Fischer, B. E.; Griesser, R.; Rheinberger, V. M.; Sigel, H.: *Z. Naturforsch. B* (1979) **34**, 208-216.
47. (a) Mitchell, P. R.; Sigel, H.: *J. Am. Chem. Soc.* (1978) **100**, 1564-1570. (b) Dubler, E.; Häring, U. K.; Scheller, K. H.; Baltzer, P.; Sigel, H.: *Inorg. Chem.* (1984) **23**, 3785-3792.
48. Lüth, M. S.; Kapinos, L. E.; Song, B.; Lippert, B.; Sigel, H.: *J. Chem. Soc., Dalton Trans.* (1999) 357-365.
49. Dvořáková, H.; Holý, A.; Masojídková, M.; Votruba, I.; Balzarini, J.; Snoeck, R.; De Clercq, E.: *Coll. Czech. Chem. Commun.* (1993) **58**, Special issue, 253-255.
50. Franchetti, P.; Abu Sheikha, G.; Cappellacci, L.; Messini, L.; Grifantini, M.; Loi, A. G.; De Montis, A.; Spiga, M. G.; La Colla, P.: *Nucleosides & Nucleotides* (1994) **13**, 1707-1719.

**Received: January 11, 2001 - Accepted: January 21, 2001 -**
**Received in revised camera-ready format: January 22, 2001**

